# When the Truth Is Out There: Counseling People Who Report Anomalous Experiences

**DOI:** 10.3389/fpsyg.2021.693707

**Published:** 2022-01-04

**Authors:** Thomas Rabeyron

**Affiliations:** ^1^Department of Psychology, Interpsy, Université de Lorraine, Nancy, France; ^2^Department of Psychology, KPU, University of Edinburgh, Edinburgh, United Kingdom; ^3^Institut Universitaire de France, Paris, France

**Keywords:** paranormal, anomalous experiences, psychotherapy, transliminality, ontological shock, spirituality

## Abstract

In this paper, we propose a clinical approach to the counseling of distressing subjective paranormal experiences, usually referred to as anomalous or exceptional experiences in the academic field. These experiences are reported by a large part of the population, yet most mental health practitioners have not received a specific training in listening constructively to these experiences. This seems all the more problematic since nearly one person in two find it difficult to integrate such experiences, which can be associated with different forms of psychological suffering. After having described briefly several clinical approaches already developed in this area, we outline the main aspects of clinical practice with people reporting exceptional experiences, in particular the characteristics of the clinician’s attitude toward the narrative of unusual events. We then present the core components of a Psychodynamic Psychotherapy focused on Anomalous Experiences (PPAE) based on three main steps: phenomenological exploration, subjective inscription and subjective integration of the anomalous experience. Such an approach, based on a non-judgmental and open listening, favors the transformation of the ontological shock that often follows the anomalous experiences into a potential source of integration and psychological transformation.

## Clinical Approaches To Anomalous Experiences

Experiences known or considered as paranormal in western culture – usually termed as anomalous or exceptional experiences in the academic field (Cardeña et al., 2014) – correspond to “experiences that are generally rare, spontaneous or provoked, involving from the subject’s point of view a non-ordinary interaction with their environment. They generate intense emotions, positive or negative, stemming from their unusual and strange aspects” (Rabeyron et al., 2010; p.634). These experiences can more precisely be classified into ten categories from a phenomenological and anthropological point of view (Rabeyron and Loose, 2015).^1^ Firstly, some of these experiences belong to a category involving an unusual “perceptive” interaction with the environment. Thus, during (1) *psi perceptions*, the person has the impression to obtain information directly from another person (telepathy), at a distance (clairvoyance) or from the future (precognition); (2) In *vision and apparition experiences*, the presence of something or someone is perceived in a quasi-hallucinatory manner and is sometimes related to a real event (for example, the death of a relative at the same time as the apparition; Fenwick and Brayne, 2011); and (3) *Out of Body Experiences* (OBE) concern a change in body awareness and lead in particular to the feeling of being situated outside of one’s body (McCreery and Claridge, 2002; Blanke and Dieguez, 2009).

From a more “projective” perspective, some people believe they can have a paranormal influence on their environment. This is especially the case in (4) *subjective psychokinesis experiences and poltergeists*, which usually involve the perceived ability to interact mentally with objects (Von Lucadou and Zahradnik, 2004) and (5) *magnetism or healing experiences*, which suppose inexplicable interactions between living being (Schmidt et al., 2004). A third category concerns “encounter” experiences in which people have the impression that they are in contact with “another world.” This includes (6) *Near-Death Experiences* (NDE; van Lommel et al., 2001; Mobbs and Watt, 2011) occurring especially after comas or clinical death, and which some people (after “seeing,” for example, a tunnel or deceased loved ones) interpret as being a journey in the afterlife (Parnia et al., 2014). A belief in life after death is also frequent in (7) *mediumistic experiences* which correspond to the alleged ability to communicate with the deceased (Taylor, 2005; Kamp et al., 2020; Elsaesser et al., 2021). In experiences of (8) *reincarnation*, the person, sometimes a child, experiment with, and believe in, past life memories (Stevenson, 1967; McNally, 2012); and (9) *Mystical experiences* can also be classified in this encounter category and correspond to an intense and global feeling of having “become one” with God or the universe. Finally, maybe the most surprising of these experiences are (10) the *abductions*, in which people are convinced that they have been abducted by aliens (Mack, 1994; Clancy et al., 2002).

Taken as a whole, approximately between one-third and half of the population reports at least one of these experiences during their lifetime^2^ (Ross and Joshi, 1992; Cardeña et al., 2014) and it is common for the same person to report several of them. Accordingly, it seems appropriate to approach and conceptualize these experiences holistically, both from a clinical and a theoretical perspective as is attempted in this paper. Several of these experiences, especially mystical experiences, have also been frequently interpreted, or even induced, by religious settings (Kripal, 2010). They are currently developing in western societies, in the context of a decrease in the influence of religions, where worldviews are more influenced by scientific and technical representations. They also frequently implicate a spiritual dimension which has been studied in particular by the field of transpersonal psychology (Tart, 2009). In this regard, Grof and Grof (1989) has proposed to consider these experiences as “spiritual emergencies,” which underlines their traumatic aspect but also their transformational potential (Kennedy et al., 1994; Ruttenberg, 2000; Palmer and Hastings, 2013).

Recent advances in several domains have provided a better understanding of these experiences. A lot of research on this topic has indeed been published in the fields of psychiatry (Lomax et al., 2011; Kamp et al., 2020), clinical psychology (Kramer et al., 2012; Roe, 2020), psychoanalysis (Eshel, 2006; Brottman, 2011; de Peyer, 2014; Si Ahmed, 2014; Reichbart, 2018), and cognitive neurosciences (Brugger and Mohr, 2008; Krippner and Friedman, 2009). A more specific approach to these experiences has also been developed by anomalistic psychology (Holt et al., 2012; French and Stone, 2013; Cardeña et al., 2014) and psi studies (Radin, 2000; Cardeña et al., 2015; Cardeña, 2018). These different lines of research, developing in complementary areas, underline that these experiences happen to people of all ages, regardless of their gender, their education, or their culture.

We will center our attention in this paper on the clinical approach of anomalous experiences. If nearly half of the persons affected consider these experiences as being pleasant, or even seek them out, the other half develop different forms of psychological and somatic suffering following them (Landolt et al., 2014; Rabeyron, 2020). Nevertheless, these experiences cannot be reduced to psychopathology, and as will be described with more details later, the relationships between anomalous experiences and mental disorders are complex and vary considerably from one person to another (Goulding, 2004a,b; Schofield and Claridge, 2007; Simmonds-Moore, 2012; Evrard, 2013). Moreover, these experiences are frequently associated with traumatic experiences, especially during childhood (Irwin, 1996), and share several characteristics with traumatic reliving as illustrated in particular by abductions (McNally et al., 2004).

The potential suffering related to these experiences can also be increased by the difficulty in talking about them due to the fear of being considered weird or crazy by relatives or medical staff (Wilde and Murray, 2010; Roxburgh and Evenden, 2016a,b). Indeed, certain aspects of these experiences suggest indeed interactions with the environment which deviate from the dominant representation of reality in western culture. Cardeña et al. (2014) have insisted on this aspect by defining anomalous experiences as “uncommon experiences (e.g., synesthesia), or those that, although they may be experienced by a significant number of persons, are believed to deviate from ordinary experience or from the usually accepted explanations of reality according to Western mainstream science” (p. 4). Consequently, many people prefer not to speak about their anomalous experiences and feel a form of culpability, or even shame, toward intimate experiences they prefer to keep secret due to their fear of not being understood.

This fear is not totally irrelevant given that most of the clinicians have not received a specific training in this domain. Thus, in a study conducted in Netherlands with 640 mental health practitioners (Corbeau, 2004), nearly half of them responded that their patients reported anomalous experiences, but four out of five of these clinicians also reported a lack of information about this topic. Similarly, academic researchers in the field of anomalous experiences report that they are ill-equipped to deal with the psychological distress that negative experiences of this kind can produce (Coelho et al., 2008). Anomalous experiences can challenge clinicians and academic researchers with narratives that may contradict their own conception of reality (Roxburgh and Evenden, 2016a). These experiences can then induce an “ontological shock” (Mack, 1994) in those who report them, but also in those who listen to them, due to a discrepancy between the reality as it was perceived before the experience and as it appeared afterward.

Consequently, specific psychological care is necessary to help people share these experiences and, more generally, we will describe how clinicians may accompany, and sympathetically support, their patients when they report such experiences. In this perspective, after a brief overview of the clinical traditions in this field, we will describe the approaches of the main counseling centers specializing in this domain. We will present in particular the approach developed at the *Center for Information, Research and Counselling about Exceptional Experiences* (CIRCEE) before proposing the main components of a Psychodynamic Psychotherapy focused on Anomalous Experiences (PPAE).

## Clinical Settings in the Field of Anomalous Experiences

There is a limited but quite long tradition of thinking about clinical counseling of anomalous experiences. It began at the end of the 19th century in the *Society for Psychical Research* in London, the *American Society for Psychical Research* in Cambridge, Massachusetts, and the *Institut Métapsychique International* in Paris. These societies were collecting these experiences in order to improve the understanding of their phenomenology (Gurney et al., 1886). It was a first step toward a clinical approach in this domain by shedding light on the necessity to account for the existence of such human experiences. A more clinical approach was developed in psychoanalytical circles who have expressed an interest in these experiences from their outset, in particular with the seminal writings of Freud, Ferenczi, and Jung about telepathy and occultism (Rabeyron et al., 2020). At this time, psychoanalysts were particularly interested in the spontaneous occurrences of telepathic phenomena shared with their analysands and catalyzed by the regression induced by the psychoanalytical setting. Other psychoanalysts have then continued to work on this topic – in particular Devereux (1953), Ehrenwald (1971), Eisenbud (1946), Servadio (1935), and Ullman (1973) – and have proposed a wider understanding of the relationships between the unconscious and these experiences.^3^ More recently, since the early 2000s, interest in psychoanalytic circles has been growing toward the clinical aspects of anomalous experiences, especially in the United States, with analysts like Mayer (2001, 2007), Eshel (2006, 2010, 2013), Totton (2003), de Peyer (2014, 2016), and Reichbart (2018).^4^ For example, Mayer (2007) explained that she “gradually had to face the realization that there were things my patients had been only half-telling me for years, things they viewed as too weird or too risky to reveal for fear that I would not believe them or – worse –would think they really were crazy” (p. 7). Consequently, these analysts question how they could, and should, be able to listen to and understand such experiences when they are reported by their analysands.

Coincident with these writings from psychoanalysts, a psychotherapeutic approach of anomalous experiences has been developed in several European countries since the 1960s.^5^ In Germany, such an approach has been developed initially by Hans Bender at the *Institut für Grenzgebiete der Psychologie und Psychohygiene* (IGPP; Bauer, 2004), in France by Hubert Larcher (Evrard, 2008) at the *Institut Métapsychique International* whose work has been pursued by the clinical psychologist and psychoanalyst Si Ahmed (2014). In Scotland, a clinic counseling service was offered with the foundation of the *Koestler Parapsychology Unit* at Edinburgh University whose main approach was based on cognitive behavior therapy with some multi-modal additions (Tierney, 2012). These first counseling services can be considered as the premise of what has been called “clinical parapsychology” (Evrard, 2007) and whose main objective is to help people to understand and cope with these experiences. In the 1980s, a few clinicians thus started to specialize in this domain and several colloquia were held about the links between clinical practice and anomalous experiences. For example, a conference has been organized about spontaneous psi cases at the University of Berkeley, in 1987, followed by a symposium entitled “psi and clinical practice” in 1989, at London, whose contributions led to a classic book in the field (Coly and McMahon, 1989).^6^

The publication of the *Varieties of Anomalous Experience* by the *American Psychological Association* (APA), in 2000, republished in 2014, has also been a founding step in this domain and represents an excellent synthesis of knowledge about anomalous experiences. The community of clinicians working on this topic has then gradually grown since the 2000s, which resulted in the first meeting of international experts on anomalous experiences in 2007, at Naarden, in Netherlands. Some of the interventions during this meeting led to a collective work intended to be a guide for clinicians confronted with these experiences (Kramer et al., 2012). Two years later, in 2009, the first conference on “Mental Health and Exceptional Experiences” was held at the University of Liverpool Hope (Simmonds-Moore, 2012). The aim of these different events was to bring together clinicians specialized in this field in order to share research and improve the quality of clinical approaches.

Currently, the main center specialized in this domain is the IGPP founded in Freiburg im Breisgau, Germany, in 1950 by Hans Bender, already mentioned, who was a medical doctor and a professor of psychology. The IGPP counseling was initially set up in collaboration with the Institute of Psychology of the University of Freiburg in 1996 (Bauer, 2004). A specific system for documenting anomalous experiences (DOKU) and a phenomenological questionnaire (PAGE; Belz-Merk, 2000; Landolt et al., 2014) have been developed there to better understand these experiences (Fach et al., 2013; Landolt et al., 2014). This institute receives about 2000 requests per year with an average of five contacts per request, i.e., an annual active file of about 500 people. Up to four clinical psychologists have worked part-time in this counseling service and the IGPP offers specific training courses recognized by the *Order of German Psychologists*.

A second organization specialized in this topic, also in Germany, is the *Scientific Society for the Advancement of Parapsychology* (*Wissenschaftliche Gesellschaft zur Förderung der Parapsychologie*), founded in Germany, in 1981, by Johannes Mischo, who was then Hans Bender’s successor to the chair of psychology and border areas of psychology at the University of Freiburg. Since 1989, particularly in response to a wave of interest in the paranormal among young Germans, the WGFP has taken a clinical orientation by developing a counseling service in the same city as the IGPP in Freiburg im Bregsau (Zahradnik, 2007). Directed by the clinical psychologist and physicist Walter Von Lucadou (1995), it has been recognized as a public utility and financed by the German government since 1991. Von Lucadou has published numerous articles and books based on data collected within the WGFP using mainly a systemic approach. His work is known for its originality from a theoretical point of view (with the Pragmatic Information Model; Von Lucadou, 1995) and for his long experience in the field of poltergeist cases (Von Lucadou and Zahradnik, 2004).

Following the example of the clinical settings developed in Germany, we have created,^7^ in 2009 in France, a teletherapy counseling service specialized in anomalous experiences: the Center for Information, Research and Counselling about Exceptional Experiences (CIRCEE, *Centre d’Information de Recherche et de Consultation sur les Expériences Exceptionnelles*). Before describing the main counseling aspects of anomalous experiences, a few words seem necessary to describe CIRCEE which is also a network that brings together French researchers and clinicians interested in anomalous experiences (psychologists, psychiatrists, philosophers, neuroscientists, etc.). The center has a Web site (www.circee.org) and most of its activity comes from its counseling service, composed currently of two clinical psychologists (working part time) and a supervisor. Since its opening in September 2009, CIRCEE has been contacted by about a thousand people and psychological counseling has been conducted with more than 750 people. More precisely, over the last 5 years – from March 2016 to March 2021 – 450 therapies have been carried out, i.e., nearly 90 therapies per year. The duration of these therapies is variable but rarely exceeds 15 sessions (45 min, weekly frequency).

Patients generally contact CIRCEE after searching the Internet about their paranormal experiences or are referred by medical doctors and clinical psychologists who have heard about the counseling service. Almost all the sessions are carried out by teletherapy (phone or zoom)^8^ since we are contacted by people who live in all parts of France and in French-speaking countries (Switzerland, Belgium, Quebec, etc.).^9^ While there might be reasons for concern that teletherapy could hinder the quality of the clinical encounter, we found, after more than 10 years of clinical practice with such an approach, that it presents as many advantages as disadvantages^10^ as confirmed recently by the development of teletherapy in other fields (e.g., teletherapy for post-traumatic stress disorder; Turgoose et al., 2018), especially since the COVID-19 crisis (Burgoyne and Cohn, 2020).

The first session usually permits to exchange with the persons about their expectations and is an opportunity for a first global description of the anomalous experiences. We also explain during this first interview how we work^11^ and what the person can expect from us. Different outcomes are possible after this first session: (1) For about half of the cases, this session is sufficient for people who find satisfactory answers to their questions about anomalous experiences and who do not wish to engage in a more in-depth analysis; (2) More rarely, the persons expectations or situation are at odds with what we propose. For example, some people might be looking for someone who could help them to develop or improve their paranormal abilities. We are also sometimes confronted with patients who are clearly describing a psychotic breakdown, with hallucinations and delirium, rather than an anomalous experience *per se*.^12^ In this type of situation, we usually suggest to the patient a reorientation toward a psychiatrist for a medical evaluation;^13^ (3) The patients are looking for a more in-depth understanding of the psychological dynamics associated with their experiences, which is usually the case when these experiences are a source of suffering. These experiences may also appear as some kind of “anomaly” or “enigma” that the person needs to better understand, opening the way for a psychological work focused initially on the anomalous experiences.

## Specificities of the Clinical Practice in the Field of Anomalous Experiences

**Figure 1:**
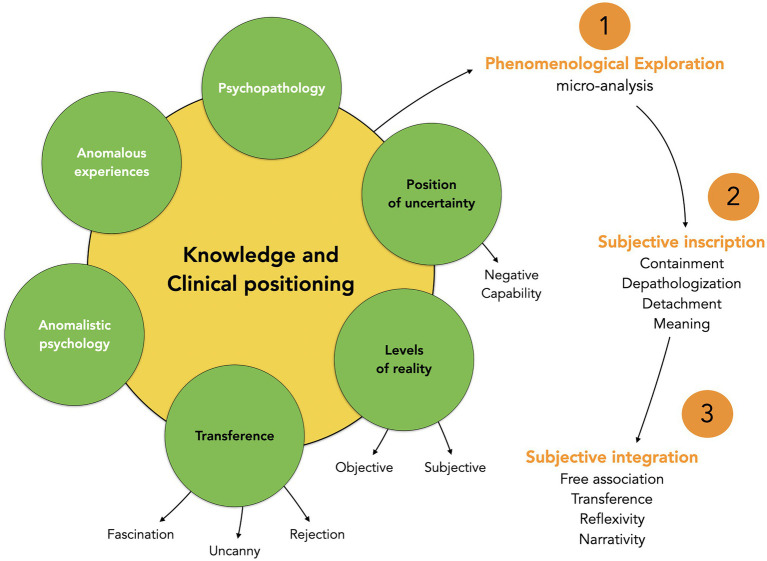
Global clinical model of counseling people who report anomalous experiences.

Before further describing the details of the psychological support proposed in the counseling service, it seems relevant to evoke briefly the type of knowledge and the clinical attitude necessary when dealing with people describing anomalous experiences as illustrated in the left circle of Figure 1.

First of all, knowledge about psychopathology is useful in order to better understand the complex relationships between anomalous experiences and psychopathology (Tierney, 1993; Wallon, 1994; Evrard, 2013, 2014). In this regard, anomalous experiences have long been a topic of debate concerning the distinction between the normal and the pathological (Evrard, 2014). Overall, the current literature shows that anomalous experiences cannot be systematically associated with psychopathology (Goulding, 2004a,b, 2005; Kerns et al., 2014). More precisely, several studies have shown a possible link between paranormal beliefs, magic thinking, and bipolar disorders (Eckbald and Chapman, 1983). People who believe in paranormal also present higher scores for hypomania and a negative relationship has been reported between belief in the paranormal and psychological coping (Irwin, 1991). Moreover, more specific psychopathological condition, like depression, have been correlated with certain exceptional experience, especially mystical experiences (Thalbourne, 1991). On the other hand, other research has failed to find a link between anomalous experiences and mental disorders (Reinsel, 2003; Goulding, 2004a,b). For example, there is no clear link between paranormal experiences and depression in other studies (Windholz and Diamant, 1974) and several studies have shown that anomalous experiences might improve mental health, wellbeing, and personal growth (Kennedy et al., 1994; Kennedy and Kanthamani, 1995).

Studies about psychopathology and anomalous experiences thus lead to contradictory results. This is particularly visible in the studies about schizotypy,^14^ a multidimensional factor of personality used to measure a tendance to psychosis (Claridge, 1997). Several researchers have indeed shown a possible link between schizotypy and anomalous experiences (Schofield and Claridge, 2007). Schizotypy is then considered, in the quasi-dimensional model (Meehl, 1990), as the expression of a mental disorder associated with anomalous experiences. But people who report such experiences usually have high levels on scales measuring unusual beliefs and perceptions, but also report low score on negative symptoms scales. Consequently, the notion of “healthy schizotype” has been proposed (McCreery and Claridge, 2002; Goulding, 2005; Mohr and Claridge, 2015) and a total model of schizotypy has been developed. In this model, people can report high scores on schizotypy scales without suffering from mental disorders and people who report anomalous experiences could then be considered as “happy schizotypes.”

**Figure 2:**
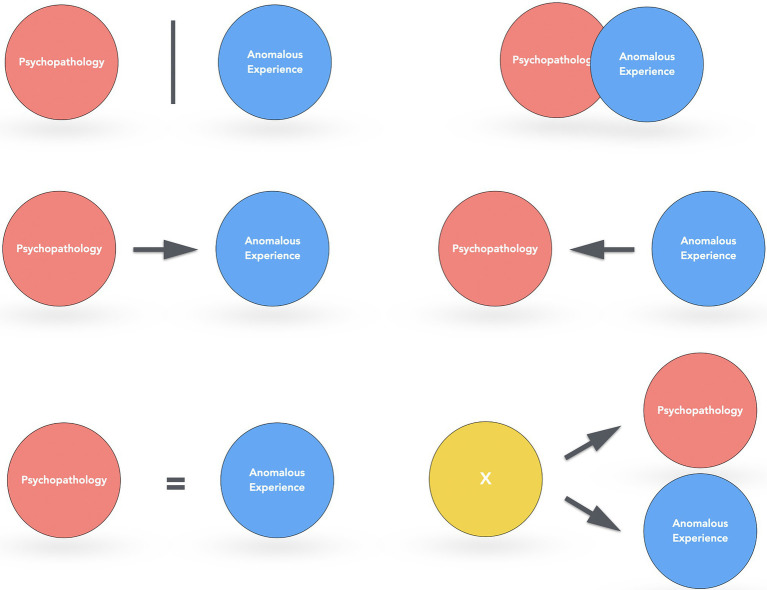
Six configurations of the relationship between anomalous experiences and psychopathology: (1) no apparent relationship between these two domains (e.g., a precognitive dream without psychopathology); (2) the anomalous experience is induced by psychopathological aspects (e.g., depression and anxiety might favor the emergence of an anomalous experience); (3) what is reported as an anomalous experience is actually a mental disorder (e.g., delirium about telepathic abilities in schizophrenia); (4) psychopathology and anomalous experiences are overlapped and they cannot easily be distinguished (e.g., certain mystical experiences during maniac episodes); (5) the anomalous experience induces psychopathology (e.g., PTSD after an abduction); and (6) another common factor favors the emergence of anomalous experiences and psychopathology (e.g., thin mental boundaries or a tendency to dissociation).

Clinical practice with people reporting such experiences underline the high diversity of configurations between anomalous experiences and psychopathology. This relationship cannot be easily reduced to a binary distinction between “normal or pathological” and probably that most screening scales for mental health and personality traits cannot render precisely the complexity of this relationship, explaining the contradictory results mentioned previously. This complexity also explains why it can be difficult for a clinician to differentiate precisely what is an anomalous experience and what is psychopathological, which can lead to misdiagnoses for clinicians who are not specialized in this domain (for example, a classical mistake is to prescribe an antipsychotic for the “treatment” of an anomalous experience).^15^ Mental health aspects associated with anomalous experiences are actually very diverse and depends on many factors (the type of anomalous experience, the personal situation of the person, the psychological structure,^16^ etc.). We can more precisely discriminate in particular six configurations concerning the relationship between anomalous experiences and psychopathology as illustrated in Figure 2. Only a detailed clinical analysis, on a case-by-case basis, can then provide a relevant analysis of the relationship between anomalous experiences and psychopathology.

Beyond these psychopathological aspects, a knowledge of the phenomenology of anomalous experiences is also useful even if it is not necessary to have had such an experience oneself. The clinician has to be able to construct a precise representation, and understanding, of the patient’s experience and should have a global knowledge of the notions, beliefs, books, known figures in the field of the paranormal. From this point of view, clinical work in this domain shares characteristics with ethnopsychiatry, as the paranormal acts as some kind of parallel world to the mainstream culture, as illustrated by the case of Paulette.^17^ This woman, in her sixties, contacted the counseling service because she was disturbed by voices that she heard on a daily basis. After losing a child when she was younger, she and her husband became interested in the paranormal and survival of life after death. They had been working with mediums in order to contact their deceased child using electronic devices. Paulette had no idea that she was going to “open a door” on her “psychic abilities.” Indeed, after a few months, she began to hear voices that she initially liked because they spoke to her about her family and reminded her about nice events. However, other voices that were more disturbing started to appear as well as strange phenomena in her home. Paulette and her husband observed unexplained movement of objects and strange behavior of household appliances. She then tried to alleviate her anxieties through relaxation and the use of an electronic device for “magnetism.” The seers, healers, and magnetizers she asked to help her mentioned that she was “affected by the lower astral.”

The voices began gradually to “get on the household appliances” and became more and more intrusive, which finally led Paulette to several hospitalizations. Psychiatrists then tell her that she was severely depressed and prescribed medication, which according to Paulette was not very helpful. She finally chose to stop these treatments even though she continued to see her psychiatrist regularly. The “world of the paranormal,” described by people like Paulette, is composed of symbols, personalities, places, and rituals that the clinician has to know about in order to improve the therapeutic alliance and the feeling of the patient that she or he is being understood. These people frequently follow a path that combines Western medicine and alternative practices (Kessler-Bilthauer and Evrard, 2018). It is therefore fairly common to see these patients after the psychiatrist, the psychologist, the hypnotist, the healer, the seer, and the exorcist who have already proposed different explanations for these experiences and how to interpret them.

Anomalistic psychology is also a useful area of knowledge to improve the understanding of the factors which may contribute to the emergence of anomalous experiences (Holt et al., 2012; French and Stone, 2013), especially the personality traits and related psychological factors (false memories, cognitive illusions, etc.) frequently associated with them (psychic permeability, fantasy-prone personality, trauma, etc.). Case studies and experimental psi research are also a useful resource concerning the more objective aspects of these experiences (Radin, 2000; Irwin and Watt, 2007; Cardeña, 2018). These different perspectives contribute to a global theoretical framework that guides the clinician’s listening. This may even sometimes concern very concrete elements. For example, the malfunction of certain household appliances – alarm clocks, microwaves, television, etc. – could induce electromagnetic waves that promote the occurrence of hallucinatory processes (French et al., 2008). The same is true for certain molds, such as *stachybotrys chartarum*, which are sometimes found in places considered as being haunted (Belkin, 2001). In some poltergeist cases, it may also be relevant to determine the source of certain noises, a classic example being the footsteps reported in the attic which are sometimes created by animals (owls, rodents, etc.) or movements in building structures due to ambient temperature change (for other factors associated with “haunted houses,” see Dagnall et al., 2020).

Beyond these three domains of expertise (psychopathology, phenomenology of anomalous experiences, and anomalistic psychology), clinical observation and clinical attitude rely on the basic ingredients of any clinical practice. Some facets of the therapeutic relationship might nevertheless be pushed to the extreme in this field, in particular transferential dynamic – the mutual and unconscious psychological influence of the patient on the therapist (Levy and Scala, 2012)^18^ – is often intense and induce reactions of fascination and rejection. Fascination because the reported situations may induce effects of sideration echoing the psychological functioning of the patient.^19^ On the contrary, rejection can take the form of disinterest, disqualification, and even irony toward the incongruity of some of these experiences. These reactions of fascination and rejection are often associated with strange feelings – more precisely what is called the “uncanny” in psychoanalysis (Allik, 2003) – due to the confrontation to the “unknown” or the “extraordinary” as illustrated by the situation of Maurice.

Maurice, a scientist in his sixties, lived through several spontaneous psychokinesis experiences which greatly disturbed him because they seemed irreconcilable with his scientific training. He described during the sessions a strange phenomenon that occurred during a scientific seminar. He was thinking about his son, worried about difficulties he encountered with him, when he found the key of his hotel room totally twisted on a table. A similar anecdote had happened to him previously with the discovery of a nail standing upright on a table at his home. He recounted with more details another experience that troubled him greatly. While enjoying a vacation with his family, Maurice took several pictures with a Polaroid camera he had just acquired. He was then in a “strange state,” thinking about a recently deceased uncle. While wondering if this uncle could see him from where he was, Maurice took a picture that profoundly upset him and his family: the picture, of his daughter, included a form in a mirror that looked like his uncle, a cigarette in his mouth, with a familiar expression. Maurice was fascinated by this picture and his father also found a striking resemblance with Maurice’s uncle, while his wife has been so scared by this supposed “paranormal photography” that she totally rejected it and eventually burned it.

Maurice could not to show this photography since it has been burned, but it is not uncommon for other patients to show photos and audio or video recording of supposed paranormal events. The usual implicit questioning from the patient is the following: “What I have experienced is real and I can prove it. Do you believe me now?.” Given that a clinical psychologist, especially in psychodynamic therapies, is supposed to be interested in everything the patient can say or show (Chouvier and Attigui, 2012), we take a look at all the “paranormal stuff” that might be proposed by the patient. We choose not to ignore this type of questioning about the reality of the events and we take into account all the different elements the persons want to share with us. Moreover, this initial step about the “objective” aspects of the experience seems necessary before the patient is able to explore the more “subjective” dimensions of the anomalous experience. The aspect is particularly important given that psychological processes are metaphorically “projected” on these objects that can be considered as a “medium” or an “interface” with the unconscious, in the same manner that a dream or a work of art can be analyzed. This can also lead to sometimes propose possible explanations concerning certain “paranormal photography” (e.g., dust, optical illusion, and flash effects). The same is true, for example, for more specific experiences like sleep paralysis^20^ for which a little information given by the clinician can make a big difference for patients frightened by what they are experiencing.

We also rely on a position of “undecidability” as proposed initially by Georges Devereux (1953) in the field of ethnopsychoanalysis. This undecidability means that the clinician has to suspend the judgment concerning the ontological dimension of the paranormal experiences. This attitude implies, for both the patient and the clinician, a “negative capability” which correspond to the capacity to tolerate doubt and uncertainty (Bion, 1965). For example, when a person considers a dream to be of a premonitory nature or describe an alien abduction experience, although the “objective” nature of the experience may be discussed, we try to not reduce the experience to what the clinician might say or think about it. Devereux (1953) explained this attitude about psi in these terms: “This is the most satisfying scientific attitude in the current state of our knowledge, but it does not simply require intellectual ingenuity in unveiling the logical characteristics that explain the nature of the link, but also a formidable capacity to tolerate frustration” (p. 32). So, even if we discuss with the patient the objective or ontological dimension of the anomalous experiences, our aim is mainly to help the patient to construct his or her own representation of the experience. This attitude seems the most appropriate as it offers a “non-judgmental space” in which the person can elaborate the meaning associated with the anomalous experience (Roxburgh and Evenden, 2016a,b). Indeed, if the clinician expresses “too much” interest in the reality of the phenomena, he risks being fascinated by the experiences and will not be able to help the patient be more objective about the potential meaning of the experience. The clinician has to be very careful from this point of view because the patients are often trying, consciously or unconsciously, to push the clinician toward a “validation” of the reality of the experience. On the other hand, if the clinician is not interested in the reality of the experiences (and, more precisely, thinks *a priori* that these experiences are impossible), the patient will be frustrated and have the feeling that the therapist is not interested in some part of his or her intimate experience (Roxburgh and Evenden, 2016a,b).

This attitude of undecidability is relatively simple to understand, but not always easy to apply in practice when we encounter situations in which we cannot understand precisely what happened to the person as illustrated by the following situation. Augustine, in her twenties, contacted the counseling service to describe an experience she had a few years ago and which she had recently discussed with the friend who was with her that day. They had gone for a walk in the woods at a picturesque site near an abbey, at a crossroads that they used to call the “no man’s land” because it was on private property. They suddenly saw “lights” and feared that it was forest rangers. They decided to get back in their car and then saw, for the duration of one minute, these lights passing in front of them. These lights were a few dozen centimeters long and appeared as “white pellets” passing over the road at a human height. Augustine thought that these lights might be related to the fact that people had been shot there during World War II. Two years later, Augustine – who had not opened up to anyone until then about this – decided to speak about this experience with her mother. The latter told her that she had a similar experience, when she was her age, in the same woods, and in a car with Augustine’s father.^21^ This narrative is a good example of “apparitions” or “visions” in which several people are involved. They are usually very disturbing for those who report them and it is often complicated to understand precisely what happened. In this regard, the clinician has to be able to handle his own frustration to not understand the nature of some of these experiences.

## Psychodynamic Psychotherapy of Anomalous Experiences

We are now going to describe more precisely how we work with people reporting anomalous experiences by proposing a model of psychotherapy – the Psychodynamic Psychotherapy of Anomalous Experiences (PPAE) – whose main principles, summarized in the right part of Figure 1, will be explained below. PPAE relies mainly on a psychodynamic approach which is described by Shedler (2010) as “a range of treatments based on psychoanalytic concepts and methods that involve less frequent meetings and may be considerably briefer than psychoanalysis proper. Session frequency is typically once or twice per week, and the treatment may be either time limited or open-ended. The essence of psychodynamic therapy is exploring those aspects of self that are not fully known, especially as they are manifested and potentially influenced in the therapy relationship” (p.98). More precisely, Blagys and Hilsenroth (2000) have described the seven most classical characteristics of psychodynamic therapies: (1) focus on affect and expression of emotion; (2) exploration of attempts to avoid distressing thoughts and feelings; (3) identification of recurring themes and patterns; (4) discussion of past experience; (5) focus on interpersonal relations; (6) focus on therapy relationship; and (7) Exploration of fantasy life. The same principles are used in PPAE but are more focused on the anomalous experiences and the feelings associated with them.

When necessary, we can also use other psychotherapeutic approaches, in particular a phenomenological approach as explained below. Indeed, the counseling of anomalous experiences, like many other clinical practices, frequently leads to theoretical and clinical integration of different techniques or theories in order to adapt as closely as possible to the situation of the patient.^22^ More specifically, we do not follow an interview grid given that the psychodynamic approach is founded on a non-directive stance allowing to follow more precisely the free associations of the patient (Rabeyron and Massicotte, 2020). Nevertheless, we can identify three main “steps” in most of the work done with the patients: (1) phenomenological exploration (how the patient describes the experience?), (2) the subjective inscription of the experience (how the patient “feels” the experience?), and (3) the subjective integration of the experience (how the patient understands the experience). We are now going to describe the main aspects of these three steps even if their length and their evolution might vary considerably from one person to another.

### Phenomenological Exploration of the Anomalous Experience: Description of the Experience

An initial phenomenological description of the experience is often relevant during the first session. The aim is to encourage the person toward an “embodied” account of the anomalous experience in a state which permits her/his to “re-experience” it from a first-person point of view (Depraz, 2014). This phenomenological exploration is also intended to help the person to get back at a sensorial and emotional level. Several methods exist to explore the phenomenological aspects of an experience and we use in particular a technique called “cognitive analysis” (Finkel, 2017) or “micro-analysis” (Rabeyron and Finkel, 2020b). This method is at the crossroad of several theories and fields of research, based on methods inspired by phenomenology (Giorgi and Giorgi, 2003) as well as introspective psychologists, like Binet, of the late nineteenth century. This method has also a number of similarities with explanatory interviews (Vermersch, 2012) and micro-phenomenology^23^ (Petitmengin and Bitbol, 2009; Bitbol and Petitmengin, 2017; Petitmengin et al., 2018), and induces a state which shares characteristics with hypnosis (Roustang, 2003; Erickson et al., 2006) and EMDR (Tarquinio et al., 2017).

If a patient is asked to describe a near-death experience, she or he might evoke the impression of having been through a tunnel at the end of which a light was perceived. This description is mainly expressed in a symbolic manner given that the patient is presenting a verbal description of the experience from memories. However, this description is a reduced and approximate account of what really happened from a subjective point of view. And the clinician also constructs an approximate representation of the patient’s experience taking the form of the representation of the tunnel described by the patient. An interview using micro-analysis leads the patient to a state of “evocation” helping him to “re-experience” the initial state at a sensory and emotional level, thus leading to a more detailed, and reliable, description of the anomalous experience. In this manner, the clinician obtains a very precise account of the experience which seems closer to what the patient has really experienced.

In order to induce this state, the person is invited first to return to the beginning or shortly before the experience (“Can you describe where you are just before the experience?”). Phenomenological questions will then be asked to guide the patient: “What do you see? What do you hear? What do you feel?.” These questions are asked in the present tense and are intended to “warm up” the memories of the experience. They are presented in terms of “how” and not “why,” so as to facilitate a concrete re-experiencing rather than a reflexive and symbolic description. The questions are kept to a strict minimum in order to reduce the impact of the suggestion: “What happens next? How do you know that…? How do you feel…?”

The clinician then carefully observes how the patient reacts. The aim is to verify, by means of different clues, that the patient is indeed re-experiencing the situation in a concrete way at sensory and emotional levels. This state of evocation will be noticeable thanks to a slowing down of the voice, a certain fixity of the gaze, eye movements that signal a search for sensory information, a phenomenological richness in the description of the experience, and the use of the present tense. The goal is thus to obtain an experience that is sufficiently close to the initial anomalous experience thanks to a precise phenomenological description, even if the intensity of the description is less than the intensity of the initial experience itself.^24^

The detailed description of the experience in this state of evocation favors its integration through an effect of reliving the representations and emotions. Indeed, the slowing down of the subjective experience helps the patient to become more “aware” of psychological elements that have gone through her or his experience in a way that was too brief to be integrated at a reflexive level. For example, certain emotions that have been cleaved from subjectivity and that were not “felt” (Winnicott, 1989). From this point of view, micro-analysis operates like the digestive system by breaking down nutrients (in this case psychological elements) in order to absorb them as already proposed by Bion (1965). The same process probably occurs with other psychotherapeutic techniques – especially hypnosis, EMDR (Calancie et al., 2018), or psychodrama (Wieser, 2007) – and relies on the principle that the recall of traumatic experiences may induces therapeutic effects (Ford, 2018; Engelhard et al., 2019).

Compared to these other approaches, cognitive analysis permits a very precise description of the patient’s representations (Finkel, 2017). Mental activity is more precisely separated into three main types of mental objects: sensations (visual, auditive, or kinesthetic), emotions (primary and secondary), and symbolic (verbal language; Rabeyron and Finkel, 2020a). These mental objects “appear” within the attentional buffer which is itself connected to a long-term information storage system. The subjective experience also relies on the attentional processes that can be present in the internal or the external world. The stream of consciousness can then be conceptualized as a cognitive algorithmic sequence – i.e., a finite sequence of internal and external states and actions – which is a synthetic representation of the successive mental states of the patient (Rabeyron and Finkel, 2020a,b).

### Subjective Integration of the Anomalous Experience: Feeling of the Experience

This phenomenological approach then opens the way to a more in-depth work of elaboration of the feelings associated with the anomalous experiences. We can identify in particular three axes of work with the patient during this step (emotional containment, de-pathologization, and detachment; see right part of Figure 1), even if it is difficult to distinguish these axes given that they often occur in parallel and that their evolution may be very different from one person to another.

#### Emotional Containment

The first axis involves the containment of the anxieties associated with the anomalous experiences while the patient frequently asks: “what has just happened to me”? In this regard, the IGPP reports that nearly 80% of the people who contact them report anxiety as a consequence of the experiences they have encountered. The “ontological shock” – initially described by the psychiatrist John Mack (1994) for alien abduction experiences – is also found after many anomalous experiences. This shock is usually the consequence of an anomalous experience which does not “fit” with the previous conception of the reality of a person (a sort of “personal paradigm of reality”). Something “impossible” has happened, which is particularly disturbing for the psychological functioning (whose main aim is to predict reality and its proprieties), leading to an overflow of the person’s descriptive capacities (Belz-Merk, 2000). These people are frequently in a crisis situation and they need some kind of reassurance as illustrated by the case Laetitia.

Aged about 30, Laetitia contacted the counseling service because she was very disturbed by phenomena occurring at the hospital where she was working as a nurse to the point that she was afraid to go there. For about 3 weeks, phenomena had been disturbing the night shifts. A door “opened by itself,” lamps lit up for no reason, and several people heard “voices, breathing and shouting.” Laetitia even had the impression, during a ward-round with one of her colleagues, that she was being chased. The most disturbing thing happened when a door she had just locked then opened while no one was in the room. Laetitia had been taking these issues very seriously, especially because this was not the first time she had been confronted to this kind of phenomenon. When she was a child, she said “objects moved” in her room and “drawers opened by themselves.” In this type of situation, the session is focused on the emotional tension associated with these experiences in order to help Laetitia to understand the nature of the experiences she reported and how she could apprehend them in a more appropriate manner. Overtly, we listen to the patient in an open and non-judgmental attitude in order to increase their ability to speak freely of an experience that was not described before due to the fear of being considered crazy. Implicitly, we tell the patient “Here, you can say everything you want, even experiences that seem impossible.” This attitude also suggests that we “take seriously” what the person is saying in order to help the expression of all the emotions linked to the experience, in particular feelings of fear, culpability, and shame, that were not expressed before.

#### Depathologizing

A second axis concerns the “de-pathologizing” of the experience. Many people experience intense fear that they are going crazy after an anomalous experience and ask: “does this experience mean I am crazy?.” This anxiety is increased by the fact that the paranormal is often stigmatized in our society as a sign of madness and this is why many people avoid talking about these experiences with a psychiatrist or a psychologist.^25^ This fear is not totally without foundation and it happens that some of these experiences are confused with or reduced to a medical condition.^26^ This pathologization of anomalous experiences can sometimes even induce a “secondary trauma” when the person attempts to share such an experience and has the feeling that it is reduced to a mental disorder (Evrard, 2007; Roxburgh and Evenden, 2016a).

Because of this, the clinician should reassure the person regarding the nature of his or her experience and support the understanding of the potential relationships between anomalous experiences and psychopathology. In this regard, we usually explain that such experiences do not mean they are becoming crazy and that many people have experienced similar things. This can seem obvious, but in certain situations, it might bring a real relief because the stigmatization of these experiences can be particularly pronounced. For example, Juliette reported frequent vision of “balls of light” both in her normal state of consciousness or on the verge of sleep. She did not know what to do with these visions which disturbed her deeply to the point that she had lost her job. She described a spiral of anguish and misunderstanding toward these experiences. An exchange with her, in a non-judgmental way, working notably on the possibility that these experiences might be the consequence of a high transliminality helped her to put words on these experiences which diminished her anxiety. She progressively found a way to cope with these experiences and regained an equilibrium state allowing her to follow her usual activities even if these visions continued.

Nevertheless, clinical work does not consist only of reassuring the person and “depathologize” the experience. As already mentioned (see Figure 2), there are many relationships between psychopathology and anomalous experiences. Consequently, the clinician has to support the patient in the recognition of the potential relationships between these factors. More precisely, two classical mistakes have to be avoided: reinforce the defensive aspect of the experience by refusing to consider what it conveys of the psychological suffering of the person (for example, consider psychotic symptoms only as the emergence of some mediumistic abilities); conversely, reduce the experience to a mental disorder (for example, the prescription of antipsychotics after an out of body experience). We try, instead, to support the person toward a better understanding of how anomalous experiences and potential psychopathology could be related as illustrated by the case of Charles.

Charles was employed as an engineer after scientific studies and had no interest in the paranormal. He then experienced “flashes” about personal events of people he could meet and started wondering if he was a psychic. He also began to practice healing and developed an interest in mediumship. Then, in a context of professional harassment, Charles experienced a period of “acute distress” which finally led to his admission to a psychiatric hospital after he thought that he was possessed and that the end of the world was coming soon. When he contacted CIRCEE, he was not sure about what he was confronted with: was it some kind of “awakening” or was he suffering from a mental disorder? Should he become a healer, a medium or should be treated as a psychiatric patient? He was torn between the conviction of having a gift – seeing events by clairvoyance, being able to heal people through magnetism – and the feeling of losing grip with reality because of some kind of amplified “sensitivity.” He also had the feeling that his thoughts were “going off the rails” and usually experienced, at these moments, a strong psychological distress associated with an intense fear of a breakdown.

Charles seemed to combine symptoms of mental disorders and anomalous experiences even if there is not clear limitation between these two domains. In this type of situation, it seems to be appropriate to help the patient to understand the relationship between these two dimensions and other psychological factors. Anomalous experiences are indeed linked to specific personality traits, like transliminality (Thalbourne, 2000), thin mental boundaries (Houran et al., 2003), absorption (Irwin, 1985), dissociation (Ross and Joshi, 1992), fantasy-prone personality (Wilson and Barber, 1983), and hypnotizability. These personality traits, which are usually highly correlated, are not pathological in themselves (Cardeña et al., 2014) but might favor the emergence of anomalous experiences and some kind of specific relation to the external and the internal worlds. We would finally have 10 sessions with Charles in order to help him to understand what could be considered as a psychotic breakdown and what could belong to the domain of anomalous experiences. He finally managed to regain a certain psychological balance and his interest in the paranormal has gradually decreased, leaving more space for existential questioning concerning the meaning of life and his professional future.

#### Detachment

Many patients are convinced that their experience is unique and particularly significant, saying something like: “what happened to me is incredible. I am unique and different!.” This uniqueness may reflect the narcissistic dimension associated with anomalous experiences, which is also frequently present in the terms used to describe them, for example, the feeling to have been “chosen” or to be an “exception” during a mystical or an abduction experience (Rabeyron, 2018). This desire to be “different” or to be “an exception” can be understood as the expression of deprivation or suffering during childhood. Certain of these people might have felt a prejudice and the anomalous experience appears, from an unconscious point of view, as a form of compensation as proposed by René Roussillon (2015) who has underlined that the “stance of exception (…) characterizes a certain form of torsion of narcissistic regulation and the relationship to the human condition” (p. 44). This psychological functioning emerging as anomalous experiences would thus express the return, through the paranormal, of narcissistic suffering. These experiences then translate a narcissistic fragility into a feeling of omnipotence which appears all the more grandiose as it supports a fragile narcissism. It is therefore difficult for the person to take a certain distance from the experience, because one of its functions is precisely to get the feeling to be different. To know without knowing, to know at a distance, to free oneself from the usual boundaries of space and time would then be the barely disguised expression of omnipotence of thoughts and magical thinking in order to control the world and avoid potential traumatic experience.

The outstanding aspects of these experiences might also induce strong negative feelings when the experience becomes terrifying (especially with abduction and poltergeist cases) and the person is not able to be objective about the experience as illustrated by the situation of Pauline. During the sessions, she described psi perceptions that “came back in droves” when she became pregnant at the same time as grieving for her grandmother. She was then embarrassed on a daily basis by “telepathic experiences” and tried to “shut it down” because she was feeling “hypersensitivity” at the surface of her skin. At this time, she also had two precognitive dreams. The first was about the Beslan bombing, involving the killing of children. She also had a dream about a terrorist attack in Spain. She then wondered if she was going crazy but she was reassured to see that this event finally happened in reality. Nevertheless, when Pauline had these dreams, she was so anxious that she did not want to leave her house. The clinical work seeks to help the patient to distance themselves from the experience, but most of the time this task is all the more complicated when the experiences are intertwined with unconscious aspects. Thus, the fact that Pauline considered these dreams as precognitive experiences, and not the potential expression of her own unconscious, prevented her from trying to understand the meaning of these experiences (In this case, dreaming of children who are killed when she is about to become a mother could be very disturbing). In this regard, these precognitive dreams might be considered as a defense mechanism (projection) that involve projecting anxieties that cannot be symbolized in the outside world.

These anxieties do not appear in delirious and hallucinatory forms but rather are expressed during dreams and hypnagogic states. Here, we observe a defense mechanism that can be found in many anomalous experiences and which is based on a projection principle: “I am not the cause of the phenomena encountered, they are the consequence of paranormal forces” or “this dream does not concern me, it is a precognitive dream.” The most intimate elements of the psychological life are thus “externalized” before being the object of denial. In Pauline’s case, a sympathetic and open listening finally helped her be more objective about her precognitive dreams and finally managed to question their meaning.

### Subjective Integration of the Anomalous Experience: Meaning of the Experience

The third step after the phenomenological exploration and the subjective inscription is the elaboration of the underlying meanings of the experience in spite of an initial attitude of many patients which might be summed up as: “this experience makes no sense.” The anomalous experience is indeed frequently described as meaningless and appears as a “foreign body” within the psyche. We usually try to question the reasons why a person reports an anomalous experience at a given moment in life. The phenomenological analysis, emotional containment, de-pathologizing, and distancing then operate as preliminary steps before the integration of the meaning of the anomalous experiences. We thus start with an “exceptional demand” which often become “more ordinary,” and therefore more “thinkable” by the patient, in order to put the anomalous experiences into perspective from a more global psychological point of view. The experience thus appears as a starting point, a doorway, from which therapeutic work can be initiated.

It then appears that the paranormal experience frequently corresponds to diverse psychodynamic functions. The clinician can help the person understand these underlying psychodynamic aspects of the experience. Each person has a different and specific relationship toward the anomalous experiences, but we can nevertheless observe some redundancies in the psychological difficulties encountered by the patients. The analysis of interviews conducted at the IGPP, using a Plan Analysis (Caspar, 2007), has shown that anomalous experiences usually correspond to five prototypes of psychological needs associated with unconscious dynamics: (1) externalizing personal difficulties (2) making life more predictable and controllable (3) regulating self-esteem (4) seeking meaning and (5) reducing emotional outbursts by avoiding painful events (Belz and Fach, 2015).

**Figure 3:**
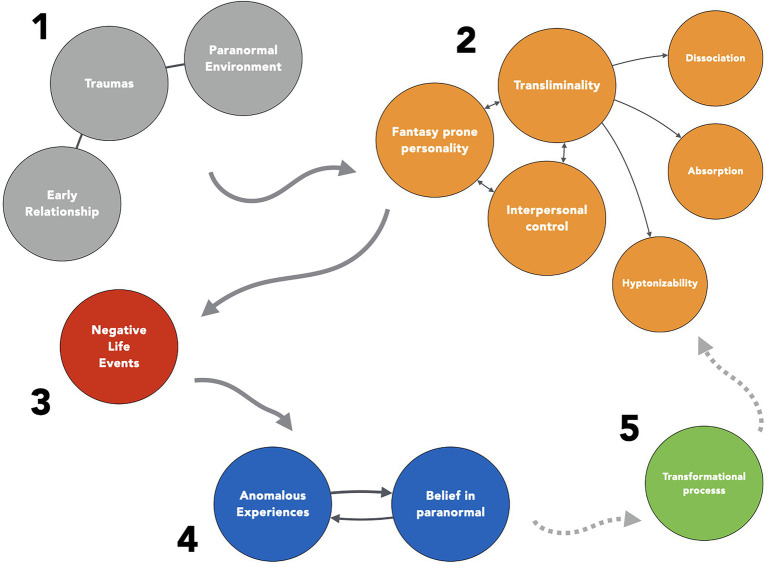
The paranormal solution.

More precisely, after many sessions with people reporting anomalous experiences, we have developed a specific model of the emergence of these experiences (Rabeyron et al., 2010; Rabeyron and Loose, 2015) taking into account childhood aspects, structure of personality, negative life events, and transformational processes as illustrated by Figure 3. Many sessions with patients reporting anomalous experiences have led us to observe that they usually appear during, or after, a strong emotional and negative even, for example an accident, a romantic break-up or the death of a loved one (fourth step in Figure 3).^27^ We have proposed to call this specific reaction a “paranormal” or “anomalous” solution (Rabeyron et al., 2010) which is usually closely related to paranormal beliefs (fifth step). There is also frequently an inaugural paranormal experience, i.e., a first anomalous experience that leads to many others and that echoes an earlier trauma during childhood as already underlined by Irwin (1996). This first step, during childhood, may correspond to a specific traumatic event, recurrent early pathological intersubjective relationship or a family environment in which the paranormal was prevailing. These different factors seem to favor an “anomaly prone personality” (Simmonds-Moore, 2012) characterized by personality traits already mentioned (second step on Figure 3: fantasy-prone personality, transliminality, interpersonal control, dissociation, absorption, and hynotizability). The fifth and last step correspond to the transformational aspects of the anomalous experience that we sometimes observer after the experience and which is often associated with an increase in wellbeing and creativity (Rabeyron, 2020).

In this model, the first and inaugural anomalous experience can be considered to be a specific coping strategy, which takes the form of an original reaction to negative life events. However, this strategy is rarely obvious to the patients, and most of the time they do not speak spontaneously about the negative life events or they do not see a connection between the anomalous event and their personal life. This model can guide the psychological counseling and help the person understand the meaning of the experience and its relationship with a broader perspective on personal life.

From this point of view, the sharing of such an experience with another person, especially a clinical psychologist, is in itself a first of step for the integration of the underlying psychological aspects of the experience and its meaning from a personal point of view. Indeed, the anomalous experience appears as an experience that needs to be shared in order to be metabolized, its sharing with another person being part of its future elaboration (Rabeyron, 2020). Such a process can be particularly beneficial when the person has the ability to elaborate psychological processes that have been “projected” into the environment as illustrated by the case of Patricia. She described how she felt unsettled by vivid premonitions she was experiencing on a regular basis when she contacted CIRCEE. These premonitions occurred especially when she was about to fall asleep, mainly in the form of symbolic expression (for example, “a torn turtleneck” or a “chest of drawers without a handle”) about her personal life and close relatives. She then needed to “translate” these dreams in order to understand their meaning. For example, “meat dreams” were, in her opinion, frequently the sign of negative future events and such dreams happened before she encountered difficulties at her job. These precognitive impressions have always been present in her life, but they were particularly pronounced a few years ago after several breakups. Patricia finally managed to elaborate these experiences and has been able to consider potential links between her personal life and the emergence of these dreams, which progressively helped her to reduce her anxiety.

The phenomenon reported by Patrick seems to be based on the same principles. He was experiencing recurrent and disturbing precognitive dreams. For example, he dreamed of a mechanical breakdown and discovered the next day that his water heater was broken. Another dream concerned two policemen he finally met afterward a few days later. He was wondering about the cause of these dreams, whether he was really perceiving the future and to what extent he himself was the cause of events that occurred later. He proposed this interesting metaphor during a session: time is similar to the backdrop of a canvas on which the “threads of the future” are grafted, giving him the impression that the future is somehow already existing. He was wondering if it was possible to change the future and was very afraid of influencing it which led him to existential questioning: “what is the point of continuing to live if the future is already written?.” Here, the metaphor of the canvas could be interpreted as a metaphor of the unconscious where the threads of the future are grafted into the canvas of the past. The fear of influencing the future might also be an expression of magical thinking and give a feeling of control in order to avoid unpleasant events.

This association between magical thinking, projection of anxieties, and precognitive experiences is also illustrated by Pascal who contacted the counseling service concerning precognitive and déjà vu experiences. It began when he was a child after his father has been electrified by a stingray while fishing. Pascal was traumatized by this event and has since been very afraid that his parents could die. In the aftermath of this episode, he experienced *déja vu* on a very frequent basis, up to five times per day. He described these as “movies” that he perceived as memories but which then really happened in real life. Pascal also reported frequent presentiment experiences. For example, he sensed that “something bad was going to happen” before he learned that a friend had an accident. But he also realized during the sessions that things were maybe more complicated than they seemed. For example, he had seen in dreams the death of a famous singer or experiencing finding treasure, neither of which occurred. Pascal finally came to the conclusion that these experiences were a mixture of forebodings, desires, and fears.

As illustrated by these different cases, the clinical work consists of helping the patient to integrate the potential meaning of these experiences and also, more generally, what might be symbolized in this manner. This implies being able, for the patient and the therapist, to “play” with the experience in order to release its symbolic potential beyond its defensive aspects. The core principles of psychodynamic therapies, already mentioned previously (see: Blagys and Hilsenroth, 2000; Shedler, 2010), are used during this process, especially the analysis of transference, free association, symbolization, reflexivity, and narrativity as illustrated on the right part of Figure 1.^28^ More specifically, we pay close attention to the transference on the clinician (how does the patient “replay” elements of his or her own history in the therapeutic relationship?), to free association (how the patients pass spontaneously from one idea to another and how associativity helps to understand the relationship between the anomalous experiences and the unconscious?), to symbolization processes (how the patients transform their experience, especially by the use of play, dream, and creativity?), to reflexivity (how the patients increase their ability to auto-represent their psychological functioning and thus manage to increase objectivity toward the anomalous experiences?), and to narrativity (how the patients can tell a coherent story of personal experience in which the anomalous experiences are integrated?). Psychotherapeutic work oriented along these different axes will favor the progressive integration of the anomalous experience. This initial topic may even become secondary and more “classical” processes might emerge during the therapy. Finally, when the underlying psychological issues require more intense or longer care, we encourage the patient to continue the therapeutic journey in a more appropriate setting for a longer psychotherapy.

## Conclusion

A significant proportion of the population report anomalous experiences and about one person in two will present difficulties in integrating them. In this regard, these “subjective anomalies” can be associated with different forms of psychological suffering and have complex relationship with psychopathology. This implies that clinicians should be trained to recognized and understand these experiences.

After having described in broad outline the type of clinical setting already developed in this domain, we have presented the clinical practice developed specifically for anomalous experiences at the CIRCEE. We have more precisely presented the leading principles of a Psychodynamic Psychotherapy focused on Anomalous Experiences (PPAE) based on three main components: phenomenological exploration (description of the experience), subjective inscription (feeling of the experience), and subjective integration (meaning of the experience). Such an approach implies general and specific knowledge (about psychopathology, anomalous experiences, and anomalistic psychology) and a specific clinical attitude (recognizing extreme forms of transference of rejection, fascination, and the uncanny; taking into account objective and subjective levels of reality; and develop an approach based on non-judgment and undecidability). These different elements lead to an open and neutral listening which favors the transformation of the ontological shock that follows some of these experiences into a potential for integration and psychological transformation.

Only a few clinicians work in counseling services specialized in this area. But the model we have proposed could also be useful for those who work in more classical clinical settings when they encounter such experiences. From this point of view, it appears that most clinicians are still not taught, or trained, to recognize these experiences (Corbeau, 2004). It follows that sometimes an attitude of rejection can develop associated with a form of stigmatization of these experiences and their reduction to mental disorders. It seems more appropriate to welcome these unusual experiences in a way that conveys to the patient that they are understood, even when these experiences challenge the therapist’s conception of reality. This attitude is all the more important in that these experiences often expressed indirectly the most intimate aspect of personality and in particular traumatic experiences. Such a clinical attitude helps the person to “go through” the ontological shock associated with some of these experiences, developing the capacity to regain a state of psychological balance and the ability to give a meaning to the experience. This process can also be an opportunity for maturation and transformational processes sharing certain characteristics with post-traumatic growth (Schubert et al., 2016). More specific studies on these transformational processes appear as a promising area of research, especially in their relationship with spiritual aspects considering their potential positive impacts on mental health and wellbeing (Brown et al., 2013; Oman, 2018; Kao et al., 2020).

Further developments in this domain could also aim at evaluating the efficacy of counseling focused on anomalous experiences. Such an approach has been initiated at the IGPP where the counseling service has been evaluated positively by the patients and led to a decrease in distress and the development of coping strategies (Landolt et al., 2014). We have also observed qualitatively that many patients seem to be relieved after the counseling sessions. The opportunity to discuss with specially trained clinicians seems to contain the downward spiral, at the somatic and psychological levels, in which some of these patients are stuck. These evaluations could rely on mental health scales, notably in the long term, in order to evaluate the evolution of the patients after the therapy. It could also help in the understanding of their evolution depending on personality traits and the type of anomalous experiences. These data could improve the coherence and the relevance of the counseling approach proposed to these patients due to a better understanding of these different aspects. This specific knowledge could also be applied more frequently in the training of clinicians in order to help them to work more effectively with their patients when such experiences emerge during the therapeutic process.

## Data Availability Statement

The original contributions presented in the study are included in the article/supplementary material, and further inquiries can be directed to the corresponding author.

## Ethics Statement

Written informed consent was obtained from the individual(s) for the publication of any potentially identifiable images or data included in this article.

## Author Contributions

The author confirms being the sole contributor of this work and has approved it for publication.

## Conflict of Interest

The author declares that the research was conducted in the absence of any commercial or financial relationships that could be construed as a potential conflict of interest.

## Publisher’s Note

All claims expressed in this article are solely those of the authors and do not necessarily represent those of their affiliated organizations, or those of the publisher, the editors and the reviewers. Any product that may be evaluated in this article, or claim that may be made by its manufacturer, is not guaranteed or endorsed by the publisher.

## Footnotes

^1^This classification is close to the categories of the *Varieties of Anomalous Experiences* (CardeÃ±a et al., 2014). Other classifications relying more on the underlying factors of these experiences have been proposed, especially a classification developed at the IGPP (for more details, see: Fach et al., 2013).

^2^There are nevertheless large differences in the frequencies of these experiences. For example, psi experiences are far more frequent than abduction or reincarnation experiences. More details about this topic in particular in: CardeÃ±a et al. (2014) and Rabeyron (2020, p. 19).

^3^This interface between psychical sciences and psychoanalysis led to a symposium coined “psi and psychoanalysis” at the first international congress of parapsychology, at Utrecht, in 1953.

^4^An annual symposium on this topic is proposed each year at the congress of the *American Psychoanalytical Association* in New York.

^5^Also, in the United States, for a recent and original theoretical framework about psi experiences relying notably on a clinical point of view, see the work of Carpenter (2012).

^6^In 1995, a symposium was also organized by the *Parapsychological Association* as part of its annual convention on therapeutic strategies concerning anomalous experiences.

^7^With Renaud Evrard – a clinical psychologist specialized in anomalous experiences and an associate professor in clinical psychology – and David Acunzo, a neuroscientist doing research in the field of anomalous experiences after a PhD at Edinburgh university.

^8^A PhD has been conducted at the IGPP on the particularities of this type of practice in the field of anomalous experiences (Fangmeier, 1999).

^9^The main exception concerns a few cases in which the sessions have been conducted at the home of the patients, which is sometimes necessary, especially for poltergeist cases. For the description of such a case, see, for example, Rabeyron (2020, p. 99).

^10^When using the phone, we do not have access to the patient’s non-verbal behavior (unless we use Zoom) which can be relevant for clinical evaluation, but we usually gain in ease of expression. Furthermore, the phone reduces the impact of the therapist demeanor which sometimes affects defense mechanisms that are frequently associated with anomalous experiences. It also allows the clinical psychologist not being in visual contact with the patient during the session which might favor free association.

^11^The person first sends a short description of the anomalous experiences by using an online form on the CIRCEE Web site. We then contact the person by email to propose a first session by phone or Zoom. At the beginning of this first session, we explain to the patient that we are clinical psychologists specialized in anomalous experiences and that we are here to understand their experiences with an open and non-judgmental approach. At the end of this first session, depending on the demand of the patient, we discuss the need and the relevance of other sessions.

^12^Like other counseling services of this type, we are indeed contacted by people who present prodromal symptoms of psychotic disorders, especially paranoid schizophrenia. These patients usually interpret manifestations of their disorders (unusual perceptions, hallucinations, influence syndrome, cognitive distortions, etc.) as having a paranormal source. The number of sessions with these patients is usually limited as they are often disappointed that we cannot confirm their paranormal abilities. Moreover, our clinical setting is most of time not suitable for such patients for whom psychiatric care, medication and hospitalization may be necessary.

^13^A patient might be experiencing a psychotic breakdown and have, at the same time, anomalous experiences. When the patient is able to describe these experiences and that we evaluate that the clinical setting of CIRCEE is safe for the patient, we may propose other sessions.

^14^See also the debates about the attenuated psychosis syndrome (Evrard and Rabeyron, 2012).

^15^This does not mean that experiences interpreted as being paranormal cannot be associated with mental disorders. For example, and as already mentioned, many patients suffering from schizophrenia have delirium concerning telepathy and influence syndrome. But it is important to not reduce all telepathic processes to this specific psychiatric condition.

^16^Using a psychoanalytical approach, we also usually take into account the predominant psychological structure of the person. There are three main structures in psychoanalytical models (neurosis, borderline, and psychosis) and the expression of the anomalous experiences will vary with these structures that can be discriminate with specific factors. For more details about these factors, see: Bergeret (1985), Maleval and Charraud (2003), Evrard (2014), and Rabeyron (2020, p. 127).

^17^Clinical case material presented in this paper has been disguised in a manner than none of the patients might be recognizable. The case of Paulette has been collected by one of the clinical psychologists of CIRCEE (Samuel CaussiÃ©). All the other clinical cases have been collected by the author of this paper.

^18^In psychoanalytical therapies, transference describes more precisely how the patient has an unconscious tendency to project feelings, desires, and expectations from past relationship to the current relation with the therapist. The transference is both a tool for understanding the patient’s psychic life and a leverage for the therapeutic process.

^19^For example, after listening to the description of a Near-Death Experience, the clinician might “share” with the patient, for a few moments, the absence of fear concerning after life due to the force of the impact of such an experience on the psyche (Rabeyron and Bergs, 2020). At the opposite, some experiences like abductions, can be very disturbing, in the same manner, induce a strong feeling of uncanny in the clinician mind. For an example of such a clinical case, see for example: Rabeyron, 2018.

^20^“Sleep paralysis involves a period of time at either sleep onset or upon awakening from sleep during which voluntary muscle movements are inhibited” (Denis et al., 2018, p. 1).

^21^In this type of situation, and as will be described later, we first listen with attention to the experience with a non-judgmental stance and by using micro-phenomenology technics. Such an approach helps the person to “re-process” the experience, even if it was not traumatic one for Augustine. It was rather some kind of anomaly that she was not able to understand, something “impossible” that happened to her. Then, we have tried to understand how Augustine was interpretating this experience in order to have a more global view of the “subjective context” of the anomalous experience. Two sessions have finally been conducted with Augustine who was not interested in doing more sessions. From a psychodynamic point of view, we had not been able to understand precisely the potential “function” of this experience. Neither did we have an “objective” explanation for what Augustine saw that night. But, sometimes, what is the most important is not that the patient has found an explanation or the meaning of the experience but rather that he or she has met someone that could listen with openness and attention to their experience. This is maybe what is the most therapeutic in the field of counseling of anomalous experiences.

^22^The technique or the theory used by the clinician is probably less important than common factors in psychotherapy as shown by many studies in the evaluation of psychotherapy (Cuijpers et al., 2019). This is probably also true for the counseling of anomalous experiences.

^23^Micro-phenomenology is a recent phenomenological approach that permit to explore very finely subjective experience thanks to specific interviews technics. It gives a very precise and details description of the inner experience.

^24^It may also happen that the clinician needs to “reduce” the intensity of the state of evocation (by the questions asked to the patient) in order to avoid the person being emotionally overwhelmed.

^25^The IGPP reports that almost half of the people who contact them already see a psychologist or a psychiatrist, which also corresponds to the data we have collected in the counseling service. They have usually chosen to not speak about these experiences with their current therapist.

^26^In the same manner than hypnosis has been considered for a long time only as a pathological state (Roustang, 2003).

^27^This link between negative life events, trauma, and anomalous experiences has been confirmed using a quantitative approach showing that people report more negative life events in the year preceding the emergence of anomalous experiences (Rabeyron and Watt, 2010).

^28^For more details on these concepts, see in particular: Roussillon, 2014.
